# Nutrition of Newborns with Hypoxic-Ischaemic Encephalopathy during Therapeutic Hypothermia - A Survey of Practice in Polish Neonatal Care Units

**DOI:** 10.34763/jmotherandchild.20242801.d-23-00115

**Published:** 2024-03-05

**Authors:** Aleksandra Warchoł, Przemko Kwinta

**Affiliations:** Department of Paediatrics, Children's University Hospital, Jagiellonian University Medical College, Cracow, Poland

**Keywords:** neonatal care, hypoxic-ischaemic encephalopathy, therapeutic hypothermia, nutrition, enteral feeding

## Abstract

**Background:**

The nutritional practice for newborns with hypoxic-ischaemic encephalopathy during therapeutic hypothermia differs among Polish neonatal care units, as no guidelines are provided. We assessed the prevailing procedures.

**Material and Methods:**

Data was collected through an anonymous, web-based questionnaire. We surveyed aspects of the current nutritional practices and the reasoning behind the choice of the feeding strategy.

**Results:**

Thirty-one responses were obtained (31/33, 94%). Based on participants’ estimations, 342 newborns are diagnosed with hypoxic-ischaemic encephalopathy and qualified for therapeutic hypothermia annually. Among them, almost ⅓ is fed exclusively parenterally, while 71% both ways—parenterally and enterally. In the vast majority of units, the introduction of enteral nutrition takes place during the first 48 hours of therapeutic hypothermia, and breast milk is primarily provided, although with substantial first feeding volume differentiation (an average of 2,9 ml/kg (0,3 - 10ml/kg)). Adverse events, such as necrotising enterocolitis, sepsis, and glycemia level disturbances that derive from the initiation of enteral nutrition, are difficult to estimate as no official statistics are provided.

**Conclusions:**

The majority of newborns after hypoxic-ischaemic encephalopathy treated with therapeutic hypothermia are fed both parenterally and enterally during the procedure, predominantly with expressed or donor breast milk. However, due to the lack of nutritional guidelines, significant variability of nutritional strategies concerning initiation time, type and volume of enteral feeds given is noted. Therefore, further studies are required to clarify feeding recommendations.

## Introduction

With an estimated prevalence of 1.5 per 1000 live births, hypoxic-ischaemic encephalopathy (HIE) remains one of the main reasons for neonatal morbidity and neurological disability in developed countries [1,2]. In terms of moderate to severe HIE, therapeutic hypothermia (TH) is a standard of care with a significant role in the reduction of death rates and central nervous system deficits, as well as providing an improvement of the neurodevelopmental outcome of the infants [3,4].

However, optimal nutrition during TH remains uncertain, and no scientific society guidelines for clinical practice are defined due to the lack of high-quality data. Therefore, feeding strategies among units worldwide vary and depend more on local experience than evidence. While in some departments parenteral nutrition (PN) is the only one provided, in others enteral feeds (EF) are introduced simultaneously. Nevertheless, recent studies have referred to the benefits of providing enteral nutrition (EN) during TH [5,6,7,8,9,10,11,12].

In Poland, Standards of Care for Newborn Health published in 2023 do not give any recommendation for feeding strategy of neonates after an episode of HIE during the cooling procedure. Thus, we conducted a survey which analyses nutrition in Polish units actively providing TH. The questions posed were related to both aspects of the prevailing practices and the reasoning behind the choice of the feeding strategy. The analysis of the former is done in the Results section of this article, while the latter is relegated to the Discussion part. To our knowledge, this is the first survey study tackling this topic in Poland.

## Material and Methods

The survey was conducted among all Polish units that provide TH to newborns after an episode of HIE. These units were identified based on the Standards of Care for Newborn Health in Poland database published in 2021. The Polish units of TH include 27 Neonatal Intensive Care and Neonatal Units and 6 Anaesthesiology and Intensive Care Children Units. Data was collected through an anonymous web-based questionnaire between April and May 2023. Thirty-one responses were obtained (31/33, 94%), and two departments did not agree to complete the survey (lack of own experiences, lack of necessary equipment to provide hypothermia).

## Results

**Figure 1.**
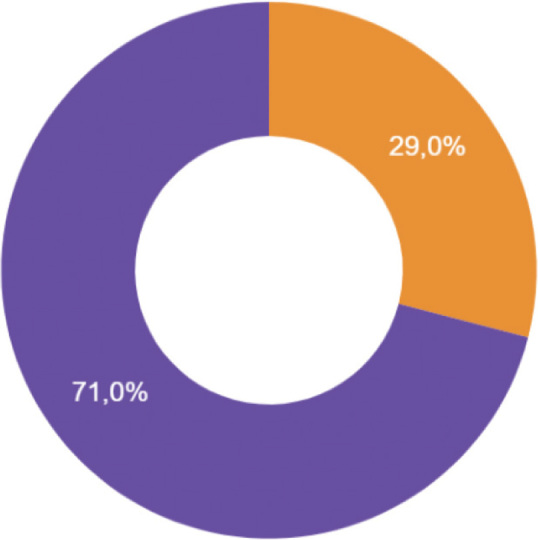
Nutrition of newborns with hypoxic-ischaemic encephalopathy during therapeutic hypothermia. Legend: Orange: Parenteral Nutrition Violet: Parenteral and Enteral Nutrition

Based on the estimations of the participants of the survey, 342 newborns are diagnosed with HIE and qualified for TH annually in the Polish units that provide the procedure actively, and during the cooling, 29% receive nihil per os and are fed exclusively parenterally, while 71% are fed both ways -parenterally and enterally, whenever no contraindications are identified (Figure 1).

### Exclusive parenteral nutrition

9 out of 10 departments that provide total parenteral nutrition (TPN) during TH introduce EF within the first 24 hours after rewarming, while one unit initiates it 24 hours after the procedure’s closure.

### Enteral nutrition

21 units in Poland (21/33; 63%) declare they provide both PN and EN to newborns receiving TH during the procedure.

Nonetheless, there is no consensus on the initiation time, type of milk, method of administration, volume of the first enteral dose of milk or assessment of food intolerance (FI) between departments.

In nine units, the introduction of EF takes place during the first 24 hours of TH, while the following nine units start EN between 24 and 48 hours of TH, three remaining units begin EN after 48 hours of TH.

Of the 21 units, eleven (52%) use exclusive mother’s own milk, eight (38%) apply mixed nutrition providing expressed or donor breast milk in addition to formula, whereas one (5%) reports providing solely donor breast milk and one (5%) only formula. Among 11 centres that use formula, eight provide a first infant formula, two a partially hydrolysed formula and one a fully hydrolysed protein formula.

In terms of the feeding method, a nasogastric tube is a predominant way of providing milk chosen by 20 (95%) of the units. The other methods are feeding by soother, used in four (19%) departments, feeding by syringe used in one (4%) department and putting milk directly on oral mucosa provided by one (4%) unit.

**Figure 2.**
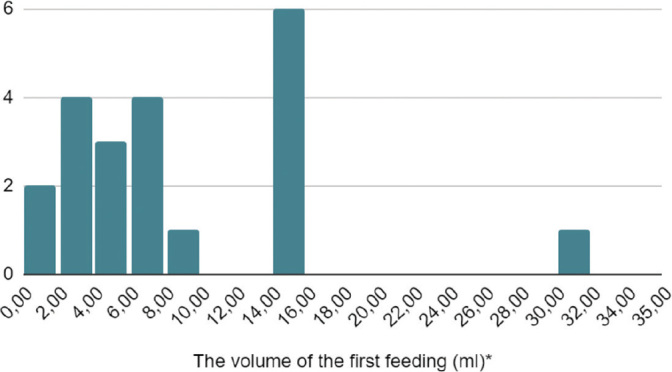
The distribution of first enteral feeding volumes (*if the body weight of a full-term newborn is assumed to be 3 kg).

The first feeding volume varies from 1 ml to 30 ml (if the body weight of a full-term newborn is assumed to be 3 kg), an average of 2,9 ml/kg (values ranging from 0,3 ml/kg to 10 ml/kg). The distribution of first EF volumes is included in Figure 2.

Food intolerance is assessed in 19 out of 21 units during TH. Food retention (18/21, 86%), vomiting occurring (15/21, 71%) and an increase in abdominal circumference (10/21, 48%) are most frequently observed.

In the vast majority of centres (19/21, 90%), the introduction of EN is not associated with the prescription of additional laboratory diagnostic tests. C-protein level, glycemia and blood gases can be assessed depending on the department in the remaining 10%.

Similarly, additional imaging tests are not routinely prescribed in 15 out of 21 units. In the other six departments, an abdomen ultrasound or X-ray is performed.

Adverse events stemming from the introduction of EN during the TH procedure, such as necrotising enterocolitis (NEC), sepsis, and glycemia level disturbances, were the subject of the last three questions of the survey. Three units were not able to estimate the frequency of NEC and glycemia metabolism disturbances, while five departments did not feel able to assess the sepsis rate. 13 centres have not observed NEC, and one department has evaluated NEC frequency at the level of 1%, while four units observed 5%. Estimated sepsis risk has ranged between 0 and 10% depending on the unit: nine departments have not reported such an adverse event, two have assessed its frequency at 1%, one at 5% and four at 10%. The occurrence of glycemia metabolism disturbances has been estimated at between 0 and 30%.

Here, we would like to stress that all the above numbers are estimates made by the respondents, and there is a lack of published official statistics of adverse events both in particular departments and in Poland as a whole.

## Discussion

Polish experience with TH spans the last 12 years, with the first application of selective head cooling (Cool-Cap) with whole-body moderate hypothermia in a newborn with features of HIE conducted in 2011 [13].

Yet, there are no Polish statistics regarding prevalence of HIE and TH procedures. According to the official data collected by the Main Statistical Department, there were 305.132 children born in Poland in 2022 [14]. Whereas, based on the respondents’ estimations, the annual hospitalisation of newborns diagnosed with HIE qualified for TH is 342. One can conclude that the estimated prevalence of HIE in Poland is about 1,1 per 1000 live births.

**Figure 3.**
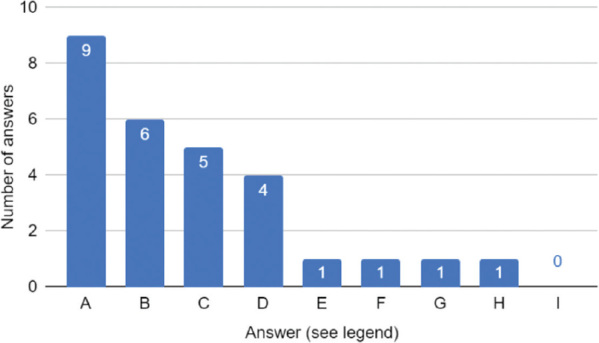
Stated reasons for withholding enteral nutrition during therapeutic hypothermia. Legend: A: Unstable hemodynamic condition of the patient B: Increased risk of NEC (Necrotising Enterocolitis) C: No recommendation for enteral feeding during therapeutic hypothermia D: Lack of safety data for enteral feeding during therapeutic hypothermia E: Use of intravenous sedation, mechanical ventilation F: Weak or absent intestinal peristalsis G: Some staff members have significant concerns regarding enteral feeding during hypothermia and are difficult to convince H: We provide trophic feeding during hypothermia if tolerated I: Increased risk of sepsis

**Figure 4.**
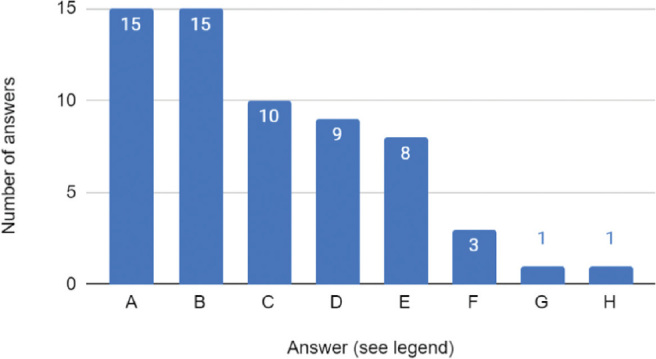
Stated reasons for introducing enteral nutrition during therapeutic hypothermia. Legend: A: Reducing the risk of sepsis B: Shortening the stay in the Neonatal Intensive Care Unit (NICU) C: Reducing the risk of NEC (Necrotising Enterocolitis) D: Increasing the percentage of breastfed newborns E: Increasing infant survival F: Gastrointestinal tract stimulation, prevention of mucosal atrophy G: Because there are no contraindications H: Gastrointestinal tract colonisation

Exclusive TPN during TH is an approach provided by ten neonatal care departments in Poland. Among them, 90% of units report the risk of instability of the hemodynamic state of the newborn as the main reason for withholding enteral nutrition. The increased likelihood of NEC is the second most common argument, listed by 60% of centres. Thus, the aim of deferring EN is to prevent gastrointestinal ischaemia reperfusion injury [15]. Moreover, half of the units rely on the absence of recommendations on providing EN during TH, while the lack of data concerning safety of EN appears in 4 out of 10 questionnaire responses. All answers are included in Figure 3. Conversely, out of 21 centres which provide feeds during TH, 15 quote reduction in the risk of sepsis and a shortage of neonatal unit stay as reasons for introduction of EN. Nearly half (10/21) of the departments declare a decrease in the frequency of NEC, nine units list higher proportion of breastfeeding at discharge and eight higher survival to discharge as arguments behind the initiation of feeds. All the reasons given for introducing EN by the departments are gathered in Figure 4.

Recent research provides some evidence favouring the introduction of EN during TH [5,6,7,8,9,10,11,12]. The study conducted by Gale et al. covered the largest cohort of 6.030 newborns, of whom almost ⅓ was fed during TH treatment, predominantly with breastmilk. The scientists found that EN was associated with a lower risk of infections, higher survival to discharge, shorter length of neonatal unit stay and a higher proportion of breastfeeding compared with the unfed group. NEC was rare in both cohorts (incidence 0·5%, [95% [CI 0·2–0·9] in the fed group vs 1·1% [0·7–1·4] in the unfed group) [5].

A systematic review and meta-analysis carried out by Kumar et al. concluded that feeding during the TH procedure did not increase the risk of NEC, hypoglycaemia or feed intolerance and might reduce the incidence of sepsis and all-cause mortality until discharge [6].

In another study, FI in hypoxic-ischaemic newborns was assessed to be frequent with no significant difference between introducing EN during or after TH [7].

Another analysis found lower inflammatory cytokine concentrations and a reduction in days of PN and hospitalisation time in the EF cohort (n=17) compared with the control group (n=17) [8].

Interestingly, the first ultrasound assessment of the gastrointestinal tract during TH revealed that blood flow velocity in the celiac artery and superior mesenteric artery remained stable during hypothermia and rose significantly after rewarming [16]. However, in this study, EF was withheld during hypothermia and introduced in the following days without an ultrasound follow-up.

In summary, however limited, the evidence has shown a potentially gastroprotective role of both TH and EF initiated during the procedure.

Analysing the questionnaire, we found a significant differentiation of nutritional strategies and reasons for pursuing them during TH in Polish neonatal units. The unification of prevailing feeding practices, however difficult (due to small groups of newborns hospitalised in particular centres and a large range of severity of illness in these infants), seems to be an urgent need. Standardising nutrition during TH by introducing uniform guidelines has the potential to improve patient outcome and ensure high-quality, consistent medical care. The latest research presented here can be a starting point for further studies aiming at developing adequate feeding procedures in Polish neonatal care units.

### Limitations of the survey

Minimal enteral feeding (MEF) was not distinguished as a separate way of providing nutrition during TH. MEF was included in the EF group.

The estimation of adverse events related to the introduction of EF during TH, like NEC, sepsis, and glucose metabolism disturbances, can be unreliable due to small groups of patients and the lack of official statistics.

## Conclusion

More than 70% of newborns after HIE treated with TH are fed both parenterally and enterally during the procedure. In light of recent studies, the introduction of EN during TH appears to be safe and feasible, as well as beneficial to neonates, after an episode of HIE. Yet, there is no agreement regarding the initiation time, type or volume of EF given. Therefore, trials, particularly randomised, intercentre, and parallel, including large cohorts of neonates, are necessary to clarify nutritional strategies for this fragile group of newborns.

### KEY POINTS

- There is a lack of high-quality data indicating optimal nutritional practices for newborns with hypoxic-ischaemic encephalopathy during therapeutic hypothermia.
- As no clinical guidelines are provided, the feeding practices differ substantially among neonatal units in Poland.
- Recent studies have noted the benefits of providing enteral nutrition during cooling; however, further research is necessary.
- Introducing uniform guidelines has the potential to improve patient outcomes and ensure high-quality, consistent medical care.
